# A thank you note to our reviewers

**DOI:** 10.1002/jgf2.505

**Published:** 2021-11-01

It would not be possible to edit the Journal without the advice of many experts. The Editor is grateful to the following people, and members of the Editorial Board, each of whom has reviewed from one to many manuscripts for the *Journal of General and Family Medicine* during the past 2 years.

Abe, Kazuhiro

Abe, Toshikazu

Ae, Ryusuke

Akaishi, Tetsuya

Akashi, Yusaku

Akita, Sadanori

Aoki, Takuya

Aoki, Yuta

Arakaki, Tatsuya

Arii, Kaoru

Asakura, Kentaro

Atagi, Kazuaki

Azehara, Atsushi

Azuma, Teruhisa

Cakiroglu, Basri

Chiba, Toshimi

Dohi, Eisuke

Endo, Takeshi

Fujieda, Yuichiro

Fujita, Kenji

Fujita, Kohei

Fukui, Sayato

Fukui, Sho

Fukushima, Kazuaki

Fukushima, Satoshi

Furukawa, Yuki

Furusawa, Haruhiko

Gao, Yanxia

Gibo, Koichiro

Gomi, Harumi

Gotoh, Jun

Gottlieb, Laura

Gurkan, Canan Gunduz

Hadano, Yoshiro

Hagiwara, Shotaro

Hagiya, Hideharu

Hamaguchi, Sugihiro

Hamano, Jun

Hamasaki, Hidetaka

Harada, Taku

Haruta, Junji

Hase, Ryota

Hashida, Nao

Hashiguchi, Teruo

Hashimoto, Kunihiko

Hashizume, Naoki

Hasunuma, Naoko

Hayase, Naoki

Hayashi, Hiroyuki

Hayashi, Tetsuro

Hayashi, Tsuneari

Himoto, Takashi

Hirai, Takuya

Hirano, Daishi

Hirasaki, Yoshiro

Hirata, Koichi

Hirayama, Yoko

Hirooka, Nobutaka

Hisada, Takeshi

Ho, Roger C

Hojo, Mariko

Homma, Yoichiro

Honda, Hitoshi

Horibata, Ken

Horikoshi, Yuho

Huh, Ji Young

Ichikawa, Hiroko

Ieda, Takeshi

Iimura, Yohei

Ikemoto, Tatsunori

Ikenaga, Yasunori

Imoto, Waki

Inoue, Kazuo

Inoue, Tatsuro

Inui, Akihiro

Irioka, Takashi

Isenberg‐Grzeda, Elie

Ishiguro, Kazuya

Ishikane, Masahiro

Ishikawa, Kazuhiro

Ishikawa, Keiichi

Ishikawa, Shizukiyo

Ishikawa, Yuki

Ishikawa, Yukiko

Ishimaru, Hiroyasu

Ito, Akihiro

Ito, Makoto

Itoh, Naoya

Iwamuro, Masaya

Iwata, Hiroyoshi

Jindai, Kazuaki

Kafeshani, Marzieh

Kaibara, Toshiki

Kajihara, Yusaku

Kakeya, Hiroshi

Kakimoto, Keisuke

Kaneko, Makoto

Kanke, Satoshi

Kanno, Keiichi

Karande, Sunil

Karimzadeh, Romina

Karli, Necdet

Kashima, Saori

Kashiwagi, Shinichiro

Katayama, Kohta

Katsuki, Naoko

Kawakami, Fumito

Kawamoto, Ryuichi

Kawasaki, Tatsuya

Kawashima, Atsushi

Kenzaka, Tsuneaki

Kikukawa, Makoto

Kinjo, Kentaro

Kinjo, Mitsuyo

Kise, Morito

Kishida, Naoki

Kita, Keiichiro

Kitahara, Takao

Kitamura, Katsuya

Kiyota, Masatomo

Kizawa, Yoshiyuki

Kobayashi, Daiki

Kobayashi, Hiroyuki

Kobayashi, Tadashi

Kohashi, Kosuke

Koike, Ryuji

Koizumi, Shunzo

Komatsu, Takayuki

Kosaka, Shintaro

Kose, Eiji

Koyama, Hiroshi

Kudoh, Rie

Kumakura, Shunichi

Kume, Yu

Kurasawa, Gotaro

Kurasawa, Miwa

Kurotori, Isaku

Kusano, Takeru

Kutsuna, Satoshi

Maeda, Keisuke

Maeno, Takami

Maeno, Tetsuhiro

Maita, Hiroki

Maki, Hiroki

Maki, Nobuyuki

Matsuda, Naoto

Matsudaira, Kei

Matsumura, Masami

Matsuo, Takahiro

Matsushita, Masahide

Matsuyama, Tasuku

Matsuyama, Yasushi

Migita, Kiyoshi

Minami, Kisei

Mineoka, Risa

Mitsuhashi, Toshiharu

Miyagami, Taiju

Miyazaki, Eishi

Miyazaki, Kei

Miyoshi, Toru

Momosaki, Ryo

Mori, Hirotake

Mori, Nobuyoshi

Mori, Takahiro

Morikane, Keita

Morishima, Ryo

Morotomi, Nobuo

Motomura, Kazuhisa

Moyama, Shota

Mukai, Tomoyuki

Murai, Kenji

Murakami, Kosaku

Muramatsu, Takashi

Murayama, Shinichi

Mutai, Rieko

Nagai, Yuki

Nagasaki, Kazuya

Nagata, Eiichiro

Naito, Toshio

Nakai, Yousuke

Nakajima, Shigemi

Nakamura, Akinobu

Nakamura, Hideki

Nakano, Toshiaki

Nakaoke, Ryota

Nakase, Hiroshi

Nakayama, Gen

Nakayama, Kuniko

Nango, Eishu

Narita, Masashi

Narumoto, Keiichiro

Nishimura, Sho

Nishimura, Takeshi

Nishioka, Hiroaki

Nishioka, Shinta

Nishisako, Hisashi

Nishizaki, Yuji

Nojiri, Shuko

Nomura, Yu

Nureki, Shin‐Ichi

Ohashi, Nobuhide

Ohira, Yoshiyuki

Ohta, Daisuke

Ohta, Ryuichi

Okada, Eijiro

Okada, Yohei

Okayama, Masanobu

Onishi, Hideki

Onuma, Yuki

Ookawara, Susumu

Osanai, Shinobu

Oshima, Kumi

Osonoi, Yusuke

Ota, Yoshiaki

Otaka, Shunichi

Otsuka, Fumio

Ozawa, Hideki

Perusquía‐Hernández, Monica

Rew, Karl

Sada, Ken‐Ei

Sada, Ryuichi

Saijo, Yasuaki

Saiki, Takuya

Sairenji, Tomoko

Saita, Mizue

Sakamoto, Yuichi

Sakata, Yasuhisa

Sakuraya, Masaaki

Saraya, Takeshi

Sasaki, Shugo

Sasaki, Yosuke

Sasao, Wataru

Sato, Juichi

Sato, Kenta

Sato, Noriko

Sato, Yuki

Satoh, Hiroaki

Satoi, Yoshinao

Sawaki, Masataka

Sawaya, Yohei

Sechi, Gianpietro

Sharma, Neeru

Shikino, Kiyoshi

Shimasaki, Teppei

Shimizu, Ikuo

Shimizu, Taro

Shiraishi, Ai

Son, Daisuke

Sugawara‐Mikami, Mariko

Sugimoto, Ken

Sugiyama, Kemmyo

Sugiyama, Yoshifumi

Suyama, Yasuhiro

Suzuki, Satoshi

Suzuki, Hideo

Suzuki, Keisuke

Suzuki, Mai

Suzuki, Norio

Suzuki, Tomoharu

Tago, Masaki

Takahashi, Fumihiko

Takahashi, Masaki

Takahashi, Noriyuki

Takamasu, Tetsuya

Takamura, Akiteru

Takayama, Shin

Takeshima, Taro

Takeuchi, Yuto

Tamaki, Hiromichi

Tanaka, Taku

Taniguchi, Toshibumi

Tanizaki, Ryutaro

Taylor, Melina

Tohara, Haruka

Tomishima, Yutaka

Toya, Yosuke

Tsukuda, Junpei

Ueda, Keigo

Uehara, Takanori

Uematsu, Haruhiro

Urushibara‐Miyachi, Yuka

VilibiÄ‐Äavlek, Tatjana

Wada, Mikio

Wakabayashi, Hidetaka

Watanabe, Yukiko

Watari, Takashi

Weissman, Charles

Wilson, Stephen

Yabuki, Taku

Yahata, Shinsuke

Yamaguchi, Yoshiko

Yamamoto, Ryohei

Yamamoto, Shungo

Yamamoto, Yu

Yamamura, Masahiro

Yamataka, Atsuyuki

Yanagisawa, Naoki

Yanai, Atsushi

Yasuda, Hideo

Yasuda, Hideto

Yeganeh, Naser

Yodoshi, Toshifumi

Yokoe, Masamichi

Yokokawa, Daiki

Yokokawa, Hirohide

Yokoyama (Toyama), Serina

Yoshida, Eriko

Yoshida, Shuhei

Yoshida, Tadashi

Yoshimi, Yusuke

Yoshimura, Yoshihiro

Zhang, Hong‐Liang

Zwaan, Laura

